# Novel Artificial Intelligence-Based Assessment of Imaging Biomarkers in Full-Thickness Macular Holes: Preliminary Data from a Pivotal Trial

**DOI:** 10.3390/jcm13020628

**Published:** 2024-01-22

**Authors:** Cesare Mariotti, Lorenzo Mangoni, Silvia Iorio, Veronica Lombardo, Daniela Fruttini, Clara Rizzo, Jay Chhablani, Edoardo Midena, Marco Lupidi

**Affiliations:** 1 Eye Clinic, Department of Experimental and Clinical Medicine, Polytechnic University of Marche, 60131 Ancona, Italyiorio.silvia@gmail.com (S.I.);; 2 Department of Medicine and Surgery, University of Perugia, S. Maria della Misericordia Hospital, 06123 Perugia, Italy; 3 Ophthalmic Unit, Department of Neurosciences, Biomedicine, and Movement Sciences, University of Verona, 37129 Verona, Italy; 4 Department of Ophthalmology, UPMC Eye Center, University of Pittsburgh, Pittsburgh, PA 15213, USA; jay.chhablani@gmail.com; 5 Department of Ophthalmology, University of Padova, 35128 Padova, Italy; edoardo.midena@unipd.it; 6 IRCCS—Fondazione Bietti, 00198 Rome, Italy; 7 Fondazione per la Macula Onlus, Dipartimento di Neuroscienze, Riabilitazione, Oftalmologia, Genetica e Scienze Materno-Infantili (DINOGMI), University Eye Clinic, 16132 Genova, Italy

**Keywords:** FTMH, artificial intelligence, OCT, IRF, SRF, EZ, ELM, HRF

## Abstract

Artificial intelligence (AI)- and deep learning (DL)-based systems have shown significant progress in the field of macular disorders, demonstrating high performance in detecting retinal fluid and assessing anatomical changes during disease progression. This study aimed to validate an AI algorithm for identifying and quantifying prognostic factors in visual recovery after macular hole (MH) surgery by analyzing major optical coherence tomography (OCT) biomarkers. This study included 20 patients who underwent vitrectomy for a full-thickness macular hole (FTMH). The mean diameter of the FTMH was measured at 285.36 ± 97.4 μm. The preoperative best-corrected visual acuity (BCVA) was 0.76 ± 0.06 logMAR, improving to 0.38 ± 0.16 postoperatively, with a statistically significant difference (*p* = 0.001). AI software was utilized to assess biomarkers, such as intraretinal fluid (IRF) and subretinal fluid (SRF) volume, external limiting membrane (ELM) and ellipsoid zone (EZ) integrity, and retinal hyperreflective foci (HRF). The AI analysis showed a significant decrease in IRF volume, from 0.08 ± 0.12 mm^3^ preoperatively to 0.01 ± 0.01 mm^3^ postoperatively. ELM interruption improved from 79% ± 18% to 34% ± 37% after surgery (*p* = 0.006), whereas EZ interruption improved from 80% ± 22% to 40% ± 36% (*p* = 0.007) postoperatively. Additionally, the study revealed a negative correlation between preoperative IRF volume and postoperative BCVA recovery, suggesting that greater preoperative fluid volumes may hinder visual improvement. The integrity of the ELM and EZ was found to be essential for postoperative visual acuity improvement, with their disruption negatively impacting visual recovery. The study highlights the potential of AI in quantifying OCT biomarkers for managing MHs and improving patient care.

## 1. Introduction

A macular hole (MH) is a full-thickness defect that occurs in the fovea. Since its initial description by Herman Knapp in 1869, various theories have been proposed to explain its development [1,2]. The pathogenesis of MH arises from the persistence of the vitreous cortex in the macular region following its separation. Vitreoretinal interface force is a significant factor in MH progression. Dynamic forces exerted by the vitreous fluid focus on the macular layers, leading to swelling in the middle and outer macular region, along with elevation and retraction of the inner retinal layers [3]. According to the cystic degeneration theory, intraretinal cystic changes occur and eventually merge, leading to the formation of a full-thickness MH [4]. The main objective of surgery for an idiopathic full-thickness macular hole (FTMH) is closing the MH and improving vision [5].

In recent years, significant advancements in surgical techniques have resulted in excellent outcomes in terms of hole closure [6]. Currently, a standard approach for vitreoretinal surgeons involves performing vitrectomy combined with internal limiting membrane (ILM) peeling and gas tamponade. The decision to proceed with surgery is often based on the patient’s reduced visual acuity or the severity of metamorphopsia reported [2].

The International Vitreomacular Traction Study (IVTS) Group has proposed OCT-based anatomical definitions and classifications for closely related vitreomacular diseases, encompassing not only FTMHs but also vitreomacular adhesion and vitreomacular traction [7].

In recent years, telemedicine and artificial intelligence (AI), especially deep learning (DL)-based systems, have made significant progress, offering promising opportunities for developing efficient tools to quantify key parameters in macular disorders [8,9,10]. Different AI-based algorithms show high performance in detecting retinal fluid [11,12] and assessing anatomical changes throughout disease progression [13]. Furthermore, AI has demonstrated its capability, particularly in eyes with age-related macular degeneration (AMD), to detect the presence of intraretinal fluid (IRF) and subretinal fluid (SRF) and to provide quantitative data in real-world scenarios [14].

The current study aimed to investigate the applicability of a validated AI algorithm for identifying and quantifying different retinal biomarkers in MHs before and after surgery. This study’s findings demonstrate that AI software serves as a dependable and consistent tool in the identification and quantification of various OCT biomarkers in eyes affected by MHs. These biomarkers are presently acknowledged as prognostic indicators, exerting an impact on the outcomes of treatment.

## 2. Materials and Methods

### 2.1. Study Design and Dataset

This retrospective study evaluated patients affected by FTMH who were surgically treated between January 2022 and June 2022 at the Eye Clinic of the University of Ancona. All the patients underwent 25-G pars plana vitrectomy with ERM/ILM peeling and gas tamponade. Blue vital dye (Twin Blue, Alchimia srl, Padova, Italy) was used for staining the ERM/ILM and the peeling was performed over an area extending up to both temporal arcades [15]. Octafluoropropane (C3F8) was employed for gas tamponade.

The exclusion criteria were as follows: MH sizes below 150 μm or above 400 μm; the affected eye having a refractive error exceeding 5 diopters; phakic patients; amblyopia, AMD, diabetic retinopathy or retinal vascular occlusion; previous intraocular surgery, excluding uncomplicated cataract surgery. A total of twenty (20) eyes from 20 patients (9 females, 45%) were included in this study.

Best-Corrected Visual Acuity (BCVA), measured as the Logarithm of the Minimum Angle of Resolution (LogMAR), was recorded at baseline and 6 months after surgery for every patient, together with a Spectral Domain Optical Coherence Tomography (SD-OCT) exam in the same timelapse. All the SD-OCT scan images were obtained using the Spectralis HRA + OCT2 platform (Heidelberg Engineering, Heidelberg, Germany). For each eye studied, a volume scan of 20 × 20° (approximately 6 × 6 mm) with 49 horizontal B-scans (with an automated real-time value set at 25 frames per scan) and a high-resolution horizontal B-scan (30° in length with an automated real-time value set at 100 frames) were analyzed by the AI algorithm at baseline and 6 months postoperatively. The minimum hole width was measured at the narrowest point of the hole in the mid-retina using the inbuilt caliper function. The measurements were performed on high-resolution horizontal scans passing through the center of the foveal depression, as a line drawn roughly parallel to the Retinal Pigment Epithelium (RPE) [6,7].

This study was conducted in accordance with the principles of the Declaration of Helsinki and written informed consent was obtained from all patients prior to study enrollment.

### 2.2. AI Algorithm Description and Analysis

In our research, we used the medical device “Ophthal” as AI (Ophthal software, Version 1.0, Mr. Doc srl, Rome, Italy, 2023). As previously described, our AI algorithm was based on adversarial generative networks, a deep learning technique [8,9,10]. It leverages a combination of labeled data (manually defined by clinicians) and a large volume of unlabeled data to create a fully labeled dataset, thereby propagating labels throughout the database. This approach falls under the category of semi-supervised learning AI, capable of self-training on pre-labeled datasets and predicting potential variations or noises that characterize these datasets, enabling effective diagnoses in real-world scenarios [16,17]. The AI software allows the user to: upload images exported from OCT software (Heyex Software Version 6.7., Heidelberg Engineering, Heidelberg, Germany). into appropriate folders; organize images by type (single scan or volumetric), execution date, examination name, physician, patient, and sequentially (follow-up); and process images by extracting significant data, both numerical (synthetic) and graphical (maps), related to both individual examinations and the differences observed between successive examinations.

**Figure 1:**
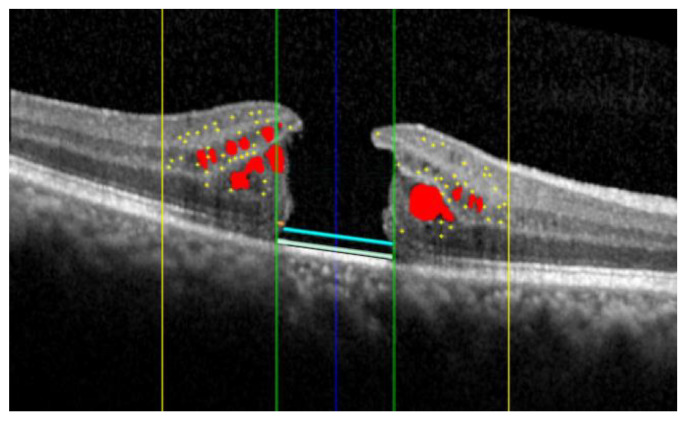

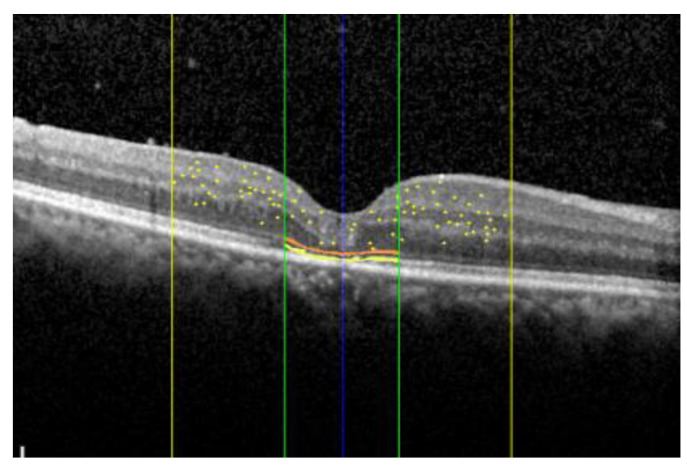
Summary of the distinct biomarkers assessed using Spectral Domain Optical Coherence Tomography (SD-OCT): intraretinal fluid (red); hyperreflective retinal foci (yellow dots) localized within the central 3 mm (yellow lines); interruption of the external limiting membrane (light blue) and ellipsoid zone (teal); the external limiting membrane (orange) and ellipsoid zone (yellow) localized within the central 1 mm (green lines).

**Figure 2:**
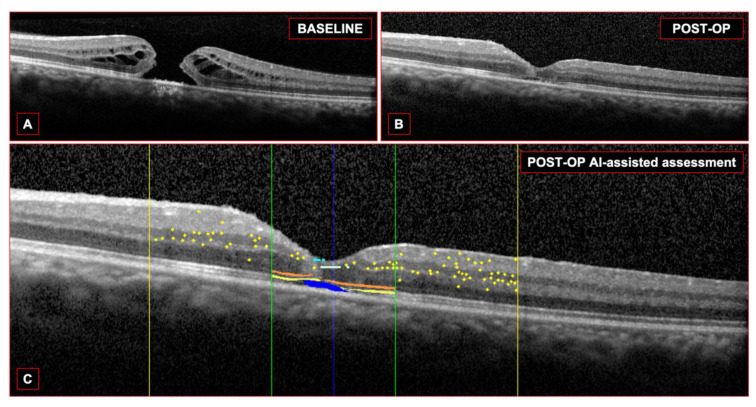
Summary of another case. The baseline OCT (**A**) and the postoperative OCT (**B**) are shown. The AI assessments of the biomarkers were overlaid (**C**): HRF (yellow dots) localized within the central 3 mm (yellow lines); ELM (orange) and EZ (yellow) localized within the central 1 mm (green lines); SRF (blue).

The AI algorithm allows for the simultaneous evaluation of several OCT biomarkers, as illustrated in Figure 1 and Figure 2.

For each eye studied, the AI automatic software was employed to segment all the OCT scans. The collected data from the entire volumetric scan included the IRF and SRF volumes. The percentage of IRF volume within specific regions was analyzed, such as the central 1 mm circle (IRF-1), the ring between 1 and 3 mm (IRF-3), and the region between 3 and 6 mm (IRF-6). The eventual presence of SRF was recorded and quantified in the postoperative scans. Additionally, the AI algorithm assessed the percentage of external limiting membrane (ELM) and ellipsoid zone (EZ) interruption within the central 1 mm of the B-scan passing through the fovea. Moreover, the number of hyperreflective foci (HRF) within the central 3 mm, as previously described, was computed in the high-resolution horizontal B-scan [18].

### 2.3. Retinal Biomarker Evaluation

The AI software included pre-trained convolutional artificial neural networks to perform the necessary functions of segmenting the OCT images of the retina. Thanks to segmentation, it was possible to identify intraretinal and subretinal fluid, hard exudates, hyperreflective foci, and different retinal layers. The segmentation conducted by the neural networks started from the intensity values of the input image pixels. These values were utilized by the first layer of the network, and the output was passed as input to the next layer, continuing until the final layer of the network. The last layer assigned to each pixel one and only one membership class among those the specific network had been trained to segment. Thus, the predictions of the membership class for each pixel formed the basis for calculating the numerical values associated with segmentations and for creating output images (masks), where pixels of different classes were colored with different colors for identification purposes.

**Figure 3:**
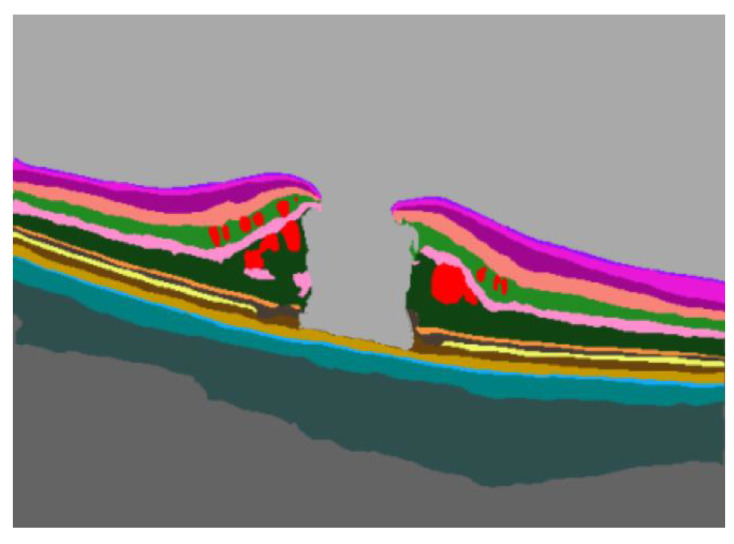

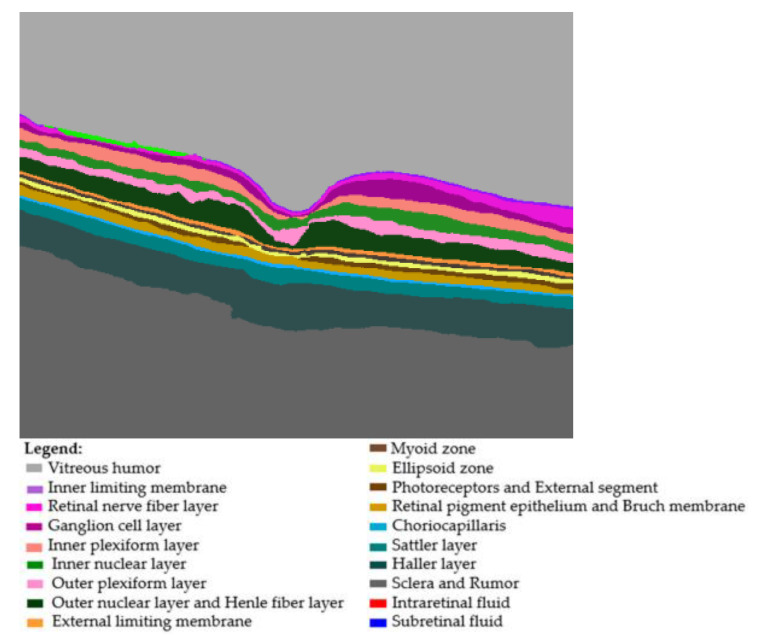
Retinal layer segmentation by the AI software.

The AI-based evaluation assessed the presence of IRF, SRF, ELM/EZ interruption, and the number of HRFs. All these factors were computed both at baseline (preoperatively) and 6 months after surgery (postoperatively). For both volumetric and linear scans, we evaluated a number of quality parameters, namely the accuracy of automated fovea centering and of the segmentation of retinal layers (Figure 3). In case of potential errors in the segmentation strategies, manual correction was allowed by the AI algorithm.

### 2.4. Statistical Analysis

The statistical analysis was performed using IBM SPSS V. 25.0.0 software. The following parameters were considered for statistical purposes: IRF, SRF, HRF, and the percentage of ELM and EZ interruption. Moreover, differences in terms of the best-corrected visual acuity (BCVA), both at baseline and 6 months after surgery, were also computed.

The quantitative data were analyzed using mean and standard deviation. The statistical tests used included the Wilcoxon non-parametric test for repeated measures and Spearman’s Rank-Order Correlation. The statistical significance level was 0.05 for all the endpoints included.

## 3. Results

Twenty (20) eyes from 20 patients (9 females, 45%) were included in this study. The mean age was 63.3 ± 13.24 years. The mean diameter of the FTMHs, measured at the narrowest point, was 285.36 ± 97.4 µm. The demographic data are summarized in Table 1.

The mean BCVA measured in LogMAR was 0.76 ± 0.16 and the median value was 0.7 (Q1 0.7-Q3 0.82) preoperatively. Postoperatively, the BCVA measured in LogMAR was 0.38 ± 0.16 and the median value was 0.38 (0.29–0.52). The difference between the preoperative and postoperative BCVA evaluations was statistically significant (*p*-value = 0.001). Thus, visual recovery was calculated as the difference between preoperative and postoperative BCVA. The mean value was 0.37 ± 0.16 and the median value was 0.36 (0.29–0.44).

The mean IRF volume, assessed using AI software, was 0.58 ± 0.63 mm^3^ preoperatively, with a median of 0.35 (0.18–0.60). Postoperatively, the mean IRF volume, assessed using AI software was 0.01 ± 0.01 mm^3^, with a median of 0.01 (0.01–0.02), with a statistically significant difference (*p* = 0.0001).

The SRF evaluation was not applicable preoperatively, as there was no detection of this item. Conversely, the mean volume of postoperative SRF identified by the AI software was 0.01 ± 0.01 mm^3^ (median 0.01 (0–0.01)).

The mean percentage of ELM interruption, detected by the AI software, was 79% ± 18% preoperatively (median value of 82% (69–94)), and 34% ± 37% postoperatively (median of 12% (0–70%)), demonstrating a statistically significant improvement after surgery (*p* = 0.006).

Similarly, the mean percentage of EZ interruption, determined by the AI software, was 80% ± 22% (median of 77% (60–99)) preoperatively and 40% ± 36% (median of 46% (17–94)) postoperatively, with a statistically significant difference between the two values (*p* = 0.007).

**Figure 4:**
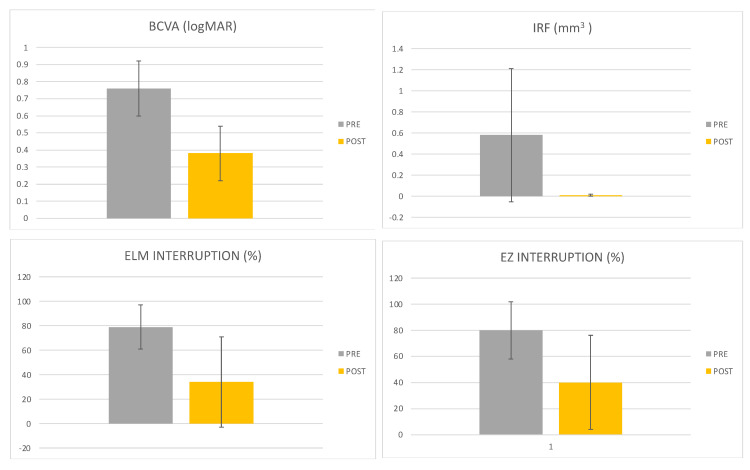
Histograms representing BCVA, IRF, and ELM and EZ interruption values in preoperative and postoperative phases.

There was no statistically significant difference (*p* > 0.05) in the number of hyperreflective foci (HRF) between the preoperative (mean of 60.86 ± 21.02; median of 66.5 (49–77.3)) and postoperative (mean of 70.79 ± 33.34; median of 71 (44–84.5)) assessments. The data are summarized in Table 2 and Figure 4.

The correlation analysis was performed using Spearman’s Rank-Order Correlation test.

A negative correlation (Spearman’s Correlation Index, R = −0.50) was found between the preoperative IRF and the recovery of BCVA after surgery, with a statistically significant value (*p* = 0.026).

Both the preoperative percentage of ELM interruption and the percentage of EZ interruption exhibited negative correlations (R = −0.50 and R = −0.53, respectively) with the recovery of BCVA after surgery. These correlations were statistically significant (*p* = 0.026 and *p* = 0.017, respectively).

A non-statistically significant correlation was found between the postoperative SRF and BCVA recovery (R = 0.05; *p* = 0.836).

**Figure 5:**
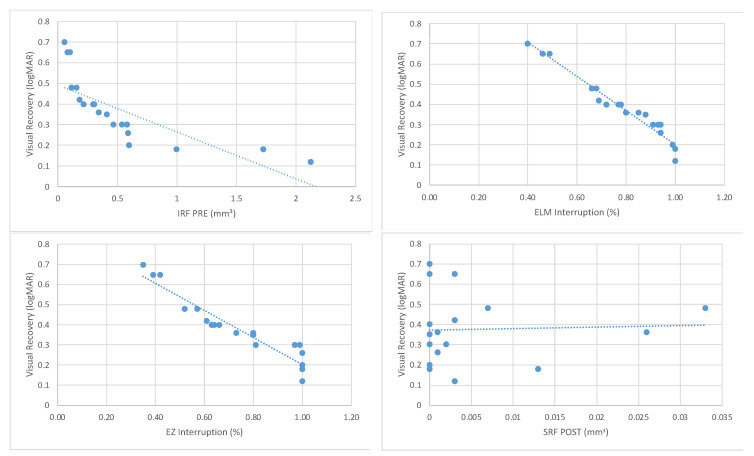
Scatter plots representing the values of the variables previously analyzed in the correlation analysis. The dashed line represents the trend line for the two parameters.

The correlation analysis data are summarized in Table 3, and the values of the variables are depicted in scatter plots in Figure 5.

Moreover, a multivariate analysis was conducted, including IRF, SRF, HRF, EZ and ELM interruption, and BCVA. The model was neither statistically significant nor predictive of visual acuity improvement; therefore, it was not presented in the results.

## 4. Discussion

The available literature indicates that AI has the potential to achieve excellent performance in identifying retinal fluid and evaluating anatomical changes during the progression of disease. Furthermore, in eyes affected by AMD, AI has proven its ability to detect the presence of IRF and SRF, both qualitatively and quantitatively, in real-world scenarios [14]. The confirmation and practical use of an AI algorithm for recognizing and measuring the predominant OCT biomarkers in diabetic macular edema (DME) have been documented [17].

The parameters that were evaluated in previous studies, after conducting a literature review using Pubmed and Google Scholar with the key words “MH, AI and OCT”, did not include IRF, SRF, ELM and EZ interruption, and HRF. Instead, they considered BCVA and hole dimensions to create a predictive model for postoperative anatomical and functional outcomes [19,20,21,22,23].

In our study, the MH width ranged from 150 to 400 μm, as per the IVTS Group classification on structural OCT [7]. All the MHs were successfully closed after surgery, with no cases of relapse. No perioperative or postoperative adverse events were reported.

As Morawski et al. stated, postoperative BCVA substantially improved in patients who showed restoration of the EZ line on OCT. Conversely, visual outcomes were poorer in cases where the EZ line was not restored [24]. Moreover, Landa et al. emphasized the importance of ELM integrity for recovery of the EZ. In their study, they highlighted that the integrity of the ELM was crucial for the restoration of the EZ line, which was considered a vital factor in achieving good postoperative visual acuity [25,26].

At the edges of the MH, the photoreceptors and other neuronal components undergo atrophic changes. This can trigger the migration of glial cells towards the developing MH, which ultimately influences the success of hole resolution after surgery. Several clinicopathological studies on repaired MHs have indicated that the movement of photoreceptors away from the central fovea, the restoration of foveal depression, and the closure of the hole following surgery are dependent on the proliferation of Müller cells, a type of glial cell [27,28]. Disarrangement in the proliferation and migration of glial cells, which are essential for closing the foveal defect, can delay the reestablishment of a continuous ELM. Consequently, this may lead to unsuccessful closure of the MH. Moreover, if Müller cells are unable to properly guide the repositioning of photoreceptors to the central fovea, the growth of normal inner and outer segments may be hindered [27,28].

Our study confirmed what previous studies have shown: the integrity of outer retinal structures, especially the EZ line, is related to better visual recovery, establishing itself as a positive predictive factor for a good postoperative outcome. Moreover, the integrity of ELM showed similar results, highlighting its central role in postoperative BCVA improvement.

In our study, for the first time, we were able to analyze EZ and ELM much more precisely, efficiently, and with an automated approach through AI software. This allowed us to calculate improvements in the EZ and ELM interruption percentage with extreme precision by comparing the integrity of these retinal layers preoperatively and postoperatively. Currently, in clinical practice, the calculation of EZ and ELM disruption is manually performed, and is therefore a time-consuming process. In contrast, the AI software simplified all calculation processes, while ensuring absolute precision and reliability.

According to previous studies, we confirmed, with an AI-based approach, the correlation between central ELM and EZ integrity and BCVA outcomes after surgery [17].

The term “hyperreflective foci (HRF)” was introduced to characterize hyperreflective lesions with a focal or dotted appearance, observed at various retinal layers using OCT imaging. Nonetheless, the exact pathological connection of HRF remains unclear, encompassing possibilities such as lipid leakage, the migration of RPE cells, the presence of macrophages/microglia, and degenerated photoreceptor cells [29].

The identification of HRF has revealed prognostic and clinical implications across multiple retinal disorders. Furthermore, understanding and defining these foci has enhanced the identification of a predominant inflammatory pathway due to glial cell activation. One of the main limitations of their use as a biomarker is the need for an automated approach to precisely quantify their numerosity [30].

In our study, artificial intelligence enabled us to accurately detect and quantify HRFs automatically. However, no statistically significant differences were observed between the preoperative and postoperative stages. Therefore, we speculate that the inflammatory pathway may not play a crucial role in the surgical outcomes of patients with FTMH. The predominantly mechanical nature of the disease associated with a minimally invasive PPV approach might be responsible for limited intraretinal glial activation or predominant migration toward the vitreoretinal surface.

For the first time in MHs, our AI algorithm has rigorously quantified the IRF in both preoperative and postoperative stages, demonstrating a significant reduction after surgery. Moreover, a higher amount of preoperative IRF showed a statistically significant negative correlation with the postoperative recovery of visual acuity. Various studies have explored the relationship between the presence of cystoid cavities and postoperative visual results, showing contradicting findings. On one side, it has been shown that the presence of IRF could be a positive prognostic factor after surgery. Brockmann et al. identified a connection between the presence of parafoveal cysts and a higher closure rate, although their analysis evaluated cysts only in a qualitative manner [31]. Chhablani et al. associated the presence of cystic edges with positive anatomical outcomes and improved final VA [32]. Although in the studies conducted by Goto et al. and Sugiura et al., cystoid cavities showed no significant correlation with postoperative BCVA, they exhibited negative correlations with preoperative BCVA and the extent of postoperative metamorphopsia [33,34]. On the other side, many studies linked IRF to a worse postoperative outcome. Joo et al. stated that cystic change is considered to be an indicator of functional retinal tissue. The improvement in visual acuity after surgery is attributed to the reduction of retinal cystic fluid, as significant functional retinal tissue remains intact [35]. Ozturk et al. conducted an analysis of cyst and MH dimensions, revealing a moderate negative correlation with postoperative BCVA [36]. Similarly, Nair et al. observed that the sizes of parafoveal intraretinal pseudocysts were linked to a lower closure rate and diminished postoperative BCVA [37]. Ruiz-Moreno et al. showed that individuals with cystic retinal changes had lower mean preoperative and postoperative VAs compared to those without such changes [38]. Our results are similar to the latter ones, showing that a major amount of preoperative IRF is related to less consistent visual recovery. The pathophysiology is still controversial, but AI could open the path for future studies on a larger number of patients in order to find precise correlations between the amount of fluid and visual function. The negative correlations between IRF, EZ interruption, and ELM interruption and visual recovery, together with the concomitant lack of significance in the difference of HRF between preoperative and postoperative phases, could underline the mechanical nature of visual impairment caused by MHs rather than an inflammatory pathway.

Furthermore, the AI software allowed us to identify the presence of SRF. Obviously, the SRF parameter was not applicable to MHs in the preoperative stage. However, in the postoperative stage, a minimal volume of SRF was identified, which did not show a statistically significant correlation with visual recovery.

Although the exact nature of SRF remains unclear, Shimozono et al. have suggested that the mere existence of SRF appears to be a regular aspect, or at least not a detrimental aspect, of the reparative mechanism in macular holes (MHs). Given that the presence of SRF could impact the thickness of the photoreceptor outer segment, a lower SRF height combined with an increased outer foveal thickness may be considered advantageous. Nevertheless, the recovery of photoreceptor outer segments can contribute to positive visual function outcomes, even in the presence of persistent SRF [39]. When the SRF volume was noticeably shallow, it was not easily discerned from the cone outer segment tips (COST) line by the AI software. The COST line is a highly reflective line detected between the EZ and the bright retinal pigment epithelium line [40]. Moreover, Govetto et al. recently investigated another specific biomarker located at this level, known as supra-RPE granular deposits, which add complexity to the morphological features of these layers [41].

The multivariate analysis performed, considering all the variables in this study (IRF, SRF, HRF, EZ interruption, and ELM interruption), did not find a model capable of predicting visual recovery after surgery. Indeed, the small number of patients included may have limited the results of this analysis. Since these are preliminary data in a pivotal trial, more comprehensive studies are needed to delve into these issues further.

In this study, the role of AI software was crucial for facilitating the quantification of IRF, SRF, HRF, and the percentage of ELM and EZ interruption. Nonetheless, the union of MH surgery and AI presents a remarkable opportunity to enhance patient care and optimize surgical outcomes.

The limitations of the current study include its retrospective nature, small simple size, and lack of long-term follow-up. This study highlights the potential of AI in quantifying OCT biomarkers for MH management and calls for further studies to explore its broader applicability in real-world settings.

## 5. Conclusions

The results of this study demonstrate that AI software is a reliable and consistent tool for identifying and quantifying different OCT biomarkers in eyes with MHs. These biomarkers are currently recognized as prognostic indicators, influencing treatment outcomes. AI software has the potential to simplify the quantification of these biomarkers in routine clinical practice in a more time-efficient manner. This study revealed a negative correlation between preoperative IRF and postoperative BCVA recovery, suggesting that greater preoperative fluid volumes may hinder visual recovery. The integrity of the ELM and EZ was found to be crucial for improving postoperative visual acuity, with their disruption negatively affecting visual outcomes. The concomitant non-statistical significance of HRF suggests the mechanical genesis of visual impairment rather than an inflammatory one. However, additional studies are required to implement this AI software in larger real-world settings, allowing for the evaluation of changes over time and clinical correlations between these changes and the progression of the disease.

## Figures and Tables

**Table 1:** Demographic data. Data referring to years and FTMH diameter are presented as mean ± standard deviation (SD).

Eyes (n)	20
Age (years)	63.3 ± 13.24
Female/Male n(%)	9 (45%)/11 (55%)
Right Eye/Left Eye n (%)	10 (50%)/10 (50%)
Mean FTMH Size/Diameter (μm)	285.36 ± 97.4

**Table 2:** Preoperative and postoperative data evaluated in this study. (N/A: not applicable).

	Preoperative			Postoperative			*p*-Value
	Mean	SD	Median (Q1–Q3)	Mean	SD	Median (Q1–Q3)	
BCVA (logMAR)	0.76	0.16	0.7 (0.7–0.82)	0.38	0.16	0.38 (0.29–0.52)	0.001
Visual Recovery (logMAR)	/	/	/	0.37	0.16	0.36 (0.29–0.44)	N/A
IRF Volume (mm^3^)	0.58	0.63	0.35 (0.18–0.60)	0.01	0.01	0.01 (0.01–0.02)	0.0001
SRF Volume (mm^3^)	/	/	/	0.01	0.01	0.01 (0–0.01)	N/A
ELM Interruption (%)	79	18	82 (69–94)	34	37	12 (0–70)	0.0006
EZ Interruption (%)	80	22	77 (60–99)	40	36	46 (17–94)	0.0007
HRF [3 mm]	60.86	21.02	66.5 (49–77.3)	60.79	33.34	71 (44–84.5)	0.9999

**Table 3:** Correlation analysis data.

Parameters Correlated	R	*p*
Preop IRF–Visual Recovery	−0.50	0.026
% ELM Interruption–Visual Recovery	−0.50	0.026
% EZ Interruption–Visual Recovery	−0.53	0.017
Postop SRF–Visual Recovery	0.05	0.836

## Data Availability

Data are fully available upon specific and motivated request to the authors.
